# Functional and Structural Characterization of Nucleic Acid Ligands That Bind to Activated Coagulation Factor XIII

**DOI:** 10.3390/jcm10040677

**Published:** 2021-02-10

**Authors:** Nasim Shahidi Hamedani, Arijit Biswas, Oliver Rudan, Rosa Tönges, Carlotta Meyring, Fabian Tolle, Günter Mayer, Johannes Oldenburg, Jens Müller, Bernd Pötzsch

**Affiliations:** 1 Institute of Experimental Haematology and Transfusion Medicine, University of Bonn, 53127 Bonn, Germany; arijit.biswas@ukbonn.de (A.B.); carlotta-meyring@web.de (C.M.); Johannes.Oldenburg@ukbonn.de (J.O.); jens.mueller@ukbonn.de (J.M.); B.Poetzsch@ukbonn.de (B.P.); 2 Department of Integrated Oncology, CIO Bonn, University Hospital Bonn, 53127 Bonn, Germany; oliver.rudan@ukbonn.de; 3 Department of Medicine 2, Hematology-Oncology Goethe University Hospital, 60590 Frankfurt am Main, Germany; rosa.toenges@kgu.de; 4 Life and Medical Sciences Institute, University of Bonn, 53115 Bonn, Germany; fabian_tolle@me.com (F.T.); gmayer@uni-bonn.de (G.M.)

**Keywords:** activated factor XIII, aptamer, SELEX, transglutaminase, aptamer modeling, nucleic acid docking

## Abstract

Coagulation factor XIII (FXIII) is a protransglutaminase which plays an important role in clot stabilization and composition by cross-linking the α- and γ-chains of fibrin and increasing the resistance of the clot to mechanical and proteolytic challenges. In this study, we selected six DNA aptamers specific for activated FXIII (FXIIIa) and investigated the functional characterization of FXIIIa after aptamer binding. One of these aptamers, named FA12, efficiently captures FXIIIa even in the presence of zymogenic FXIII subunits. Furthermore, this aptamer inhibits the incorporation of FXIII and α2-antiplasmin (α2AP) into fibrin(ogen) with IC_50_-values of 38 nM and 17 nM, respectively. In addition to FA12, also another aptamer, FA2, demonstrated significant effects in plasma-based thromboelastometry (rotational thromboelastometry analysis, ROTEM)-analysis where spiking of the aptamers into plasma decreased clot stiffness and elasticity (*p* < 0.0001). The structure–function correlations determined by combining modeling/docking strategies with quantitative in vitro assays revealed spatial overlap of the FA12 binding site with the binding sites of two FXIII substrates, fibrinogen and α2AP, while FA2 binding sites only overlap those of fibrinogen. Taken together, these features especially render the aptamer FA12 as an interesting candidate molecule for the development of FXIIIa-targeting therapeutic strategies and diagnostic assays.

## 1. Introduction

Coagulation factor XIII (FXIII) is a protransglutaminase consisting of two B subunits (FXIII-B) which act as carrier for two A subunits (FXIII-A) in plasma [1]. FXIII in plasma is activated by thrombin-mediated cleavage of an N-terminal activation peptide (residues 1–37) from the A subunit and dissociation of the transglutaminase FXIII-A dimer (activated FXIII [FXIIIa]) from the B subunits in the presence of calcium [2]. Non-activated plasma FXIII is bound to fibrinogen via FXIII-A subunits and the binding is unaffected by fibrinogen polymerization [3]. Using several antibodies specific to various regions of fibrinogen, the binding region of FXIII-A on fibrin was localized by Procyk et al., who suggested the fibrinogen αC residues (Aα389-402) as FXIII-A binding site [4]. Activated FXIII plays an important role in clot stabilization and composition by cross-linking the α- and γ-chains of fibrin [5] and cross-linking of α2-antiplasmin (α2AP) to fibrin, resulting in increased resistance of the clot to mechanical and proteolytic challenges [6]. It has been reported that elevated FXIII levels in female correlate with angiographically proven coronary stenosis (CS) and with the history of myocardial infarction (MI), suggesting the influence of higher FXIII plasma levels in the acute thrombotic events rather than the development of atherosclerotic plaques [7,8]. Similarly, elevated FXIII increased the risk of peripheral arterial disease (PAD) in women [9].

The clot-stabilizing function and downstream functionalities of FXIIIa make FXIII a favorable target for drug development since specific inhibitors targeting FXIII would neither interfere with thrombin generation nor with fibrin formation and platelet activation. Furthermore, currently available anticoagulants such as direct thrombin- or FXa-inhibitors are associated with the life-threatening side effect of internal bleeding, particularly intracranial, gastrointestinal, and retroperitoneal bleeding [10,11,12,13]. Thus, efforts to develop new approaches to safely prevent thrombosis are of substantial clinical impact. 

FXIII inhibition might be achieved by direct interaction of small molecule inhibitors with the active site or modulation of the enzyme activity via allosteric binding sites. In the first case, the accessibility of the active site is limited through the reversible or irreversible blockage of the catalytic triad by inhibitor molecule [14,15]. Another approach is influencing the activation mechanisms of FXIII. Lukacova et al. derived a monoclonal antibody, MAb 309, against a peptide sequence in the thrombin activation site of factor XIII which inhibits 99% of apparent FXIII activity in a standard transglutaminase assay [16]. Besides MAb 309, the antibodies MAb 9C11 and MAb 10G10 were also found to affect the thrombin-mediated FXIII activation pathway [17]. Although these antibodies block FXIII activation through blockade of the thrombin binding site or induce conformational changes in FXIII molecular structure which leads to the inhibition of FXIII activation, due to the competitive mechanism of binding kinetics, an excess amount of thrombin causes a displacement of the antibodies, which limits the usage of this kind of antibodies for pharmacological purposes [18]. The inhibition of fibrin crosslinking via amine-containing competitive inhibitors which act as alternative substrates for the FXIIIa-catalyzed reaction is another approach to address FXIII activity [19]. However, such competitive substrates do not only interact with FXIIIa but also inhibit other transglutaminases.

Aptamers are single-stranded DNA or RNA oligonucleotides which bind with high affinity and specificity to their target molecules via non-covalent binding. The extended area of recognition and binding of aptamers in comparison to antibodies enable aptamers to discriminate between two structurally similar molecules. This ability is of high importance where small changes in protein sequence such as cleavage of a small activation peptide or structural changes without protein sequence alteration might change the function of a protein. Several aptamers with the ability to distinguish between the active and inactive state of a protein are reported [20,21]. A series of DNA aptamers which were selected against activated protein C (APC) show substantially lower affinity to the zymogenic protein C in comparison to the active enzyme, APC [20,22]. Furthermore, a G-quadruplex forming aptamer specific for thrombin exhibited reduced affinity to the zymogen prothrombin when compared to its binding to thrombin [21,23]. Another advantage of aptamers is that rationally designed short oligonucleotides complementary to the aptamer sequence can act as antidotes to efficiently reverse the aptamer activity. Hence, aptamers can serve as regulable therapeutics accessible by rationally designed complementary sequences [24,25]. 

In this study, we selected and characterized a series of DNA aptamers that bind to activated FXIII (FXIIIa). We demonstrate that one of these aptamers, FA12, warrants further functional studies to fully reveal the diagnostic as well as therapeutic potentials of this molecule. 

## 2. Experimental Section

### 2.1. Materials

All basic chemicals were purchased from Merck/Sigma-Aldrich (Darmstadt, Germany) unless otherwise noted. The randomized single-stranded (ss) DNA library IHT1 (5′-AAG CAG TGG TAA GTA GGT TGA—N40 (25% each A/G/C/T)—TCT CTT CGA GCA ATC CAC AC-3′ was synthesized and PAGE-purified by Microsynth (Balgach, Switzerland). IHT1-amplification primers and 5′-biotinylated capture molecules for the IHT1 library (5′-Biotin-GTG TGG ATT GC-3′) were synthesized and HPLC-purified by Eurogentec (Seraing, Belgium). All individual aptamers FA1, FA2, FA3, FA6, FA8 and FA12, as well as the negative control oligonucleotide (5′-TAC TGT CAC GAG GAT ATA GCA CAT TAG TTC AGA TAC GAT TGT TAC TGT CA-3′) were ordered either without or with 5′-biotin modification from Ella Biotech (Martinsried, Bavaria, Germany). FXIII-assay fluorogenic substrate, Abz-NE(CAD-DNP)EQVSPLTLLK-OH, coagulation factor XIII purified from human plasma (pFXIII), A subunit of FXIII (FXIII-A), B subunit of FXIII (FXIII-B), activated FXIII (FXIIIa), and Tridegin were purchased from Zedira (Darmstadt, Germany). Peroxidase-conjugated goat anti-human α2-antiplasmin (α2AP) antibody was purchased from Affinity Biologicals (Ancaster, Canada). The BM chemiluminescence substrate (POD) was purchased from Roche (Mannheim, Germany). The Berichrom^®^ FXIII assay kit and recombinant human tissue factor (Dade^®^ Innovin^®^) were obtained from Siemens Healthcare Diagnostics (Marburg, Germany). Hemocomplettan^®^P was purchased from CSL Behring (Marburg, Germany). Corn trypsin inhibitor (CTI) was purchased from Haematologic Technologies (Essex Junction, VT, USA). Argatroban was bought from Mitsubishi Pharma Deutschland GmbH (Düsseldorf, Germany). Ultrapure streptavidin was purchased from AppliChem (Darmstadt, Germany). Actilyse (Tissue-type plasminogen activator, tPA) was purchased from Boehringer Ingelheim (Biberach, Germany).

### 2.2. Formulations of Used Buffers

Capillary electrophoresis selection buffer: 25 mM Tris-HCl, 30 mM NaCl, 1 mM KCl, 1 mM CaCl_2_, 1 mM MgCl_2_ pH 8.3; Binding buffer: D-PBS-buffer (containing 0.9 mM CaCl_2_ and 0.5 mM MgCl_2_ in the 1× concentrated solution) was purchased as a 10× concentrate at a pH of 5.3 from Sigma (cat. no. D1283) and the pH was adjusted to 7.4 during preparation of the 1x concentrated buffer and 0.1% bovine serum albumin (BSA) was added; Assay buffer: 50 mM Tris-HCl, 100 mM NaCl, 2 mM CaCl_2_, 2.5 mg/mL BSA, pH 7.4; Substrate buffer: 50 mM Tris-HCl, 100 mM NaCl, 2 mM CaCl_2_, 100 mM H-Gly-Ome*HCl, 2.5 mg/mL BSA, pH 7.4; Washing buffer: 1 × PBS, pH 7.4, 1 mM each MgCl_2_/CaCl_2_, 0.05% Tween 20; Coating buffer: 30 mM Na_2_CO_3_, 200 mM NaHCO_3_, pH 9.0; Blocking buffer: 1× PBS, pH 7.4, 20 mg/mL BSA, 0.05% Tween 20.

### 2.3. Capillary Electrophoresis-Systematic Evolution of Ligands by Exponential Enrichment (CE-SELEX)

CE-SELEX was performed using a ProteomeLab PA 800 (Beckman Coulter Inc., Fullerton, CA, USA) as previously described [26]. In brief, six cycles of CE-SELEX against recombinant human FXIIIa were performed using the ssDNA-library IHT1. Generation of ssDNA for subsequent selection cycles was done by the Capture and Release (CaR) procedure using biotinylated IHT1 capture molecules as previously described [27]. The starting concentration of FXIIIa of 1 µM was reduced with each selection cycle to incrementally increase the stringency of the selection.

### 2.4. Next Generation Sequencing (NGS) 

NGS was performed using sequencing by synthesis technology on a HiSeq 1500 instrument (Illumina, San Diego, CA, USA). The TruSeq DNA PCR-Free (LT) sample preparation kit (Illumina) was used for adapter ligation. A detailed description of the sample preparation protocol has been previously published [28]. Data processing of the raw sequencing data was done by AptaIT (Munich, Germany) using the COMPAS software.

### 2.5. Determination of Dissociation Constants

Biolayer Interferometry (BLI) technology was applied to determine the binding kinetics of all six aptamers to recombinant FXIIIa, FXIII-A, and pFXIII. A BLItz system (Pall Life Sciences, Dreieich, Germany) and the BLItz 1.2 software were used for corresponding analysis. Briefly, a High-Precision Streptavidin (SAX) Biosensor (Pall Life Sciences) was loaded for 120 to 200 s with each 3′-biotinylated aptamer (1 µM). Then, the loaded biosensor was equilibrated in binding buffer for 30 s followed by immersing in a drop containing different concentrations of target protein or a tube of 500 µL of binding buffer to determine the association and dissociation rate constants, respectively. All measurements were performed at a shaking speed of 2200 rpm.

### 2.6. Determination of the Impact of Aptamer Binding on Isopeptidase and Transglutaminase Activity of FXIIIa

The effect of aptamer binding on the isopeptidase activity of FXIIIa was assessed using the fluorogenic peptide substrate Abz-NE(CAD-DNP)EQVSPLTLLK-OH. FXIIIa (12.8 nM, equal to 1 µg/mL) was incubated with increasing concentrations of each aptamer (0.32 nM–3.16 µM) in assay buffer for 30 min followed by transferring of 20 µL of the mixture to white F8 Fluoronunc modules (Thermo Fisher Scientific, Wiesbaden, Germany). Subsequently, 80 µL of 25 µM fluorogenic substrate in substrate buffer was added and substrate hydrolysis rates were recorded at λ_ex_ 310 nm and λ_em_ 420 nm using a Synergy 2 microplate reader (BioTek, Bad Friedrichshall, Germany).

The effect of aptamer binding on the transglutaminase activity of FXIIIa was quantified using the Berichrom^®^ FXIII assay kit. Briefly, FXIIIa (4 nM) was incubated for 30 min with different concentrations of each aptamer (0.32 nM–3.16 µM) in 20 µL of assay buffer followed by addition of 80 µL of reagent mixture containing a 1 + 1 mixture of activator reagent (10 IU/mL bovine thrombin, 2 mg/mL Gly-Pro-Arg-Pro-Ala-amide, 1.2 mg/mL CaCl_2_, 10 mg/mL hexadimethrine bromide, 0.5 mg/mL NADH and bovine albumin in 100 mM BICINE buffer; pH 8.3) and detection reagent (20 IU/mL GLDH, 2.4 mg/mL synthetic peptide as FXIII substrate, ADP, 1.4 mg/mL glycine ethylester, 2.7 mg/mL α-ketoglutarate, bovine albumin in 10 mM HEPES buffer; pH 6.5). FXIIIa reacts with the synthetic substrate to release ammonia which in turn oxidized NADH to NAD. The reduction in the absorbance due to the reduction of NADH amount was measured at 340 nm using the Synergy 2 microplate reader.

### 2.7. Determination of the Capturing Capacity of the Aptamers

To determine the capacity of aptamers in capturing FXIIIa, streptavidin-coated white F8 FluoroNunc modules (Thermo Fisher Scientific, Wiesbaden, Germany) were loaded with 3′-biotinylated aptamers (100 nM solution) as described before [22]. Subsequently, 100 µl of 30 nM FXIIIa in binding buffer was incubated in the wells for 30 min. Wells were washed 3 times with washing buffer using an automated plate washer (ELx50, Biotek, Bad Friedrichshall, Germany). Captured FXIIIa was quantified after addition of 100 µL of 25 µM FXIIIa-specific fluorogenic substrate using the Synergy 2 microplate reader. 

In order to determine the interfering effect of structurally or functionally relevant molecules on FXIIIa capturing by aptamers, initially maxisorb microtiter plates were coated with 10 µg/mL bovine serum albumin (BSA)-biotin (100 µL/well) in coating buffer at 4 °C overnight followed by washing with washing buffer. Subsequently, streptavidin (10 µg/mL, 100 µL/well) was added to the wells and incubated for 1 h at RT. After washing, the wells were blocked using 200 μL/well of blocking buffer for 2 h at RT, the remaining blocking buffer was aspirated and 3′-biotinylated aptamers were loaded (100 nM, 100 µL/well). FXIIIa (30 nM) was mixed with increasing concentrations of FXIII-A, FXIII-B, or pFXIII (1–100 nM) and 100 µL of these mixtures were transferred into the streptavidin-coated wells primed with 3′-biotinylated aptamers. After 30 min of incubation, wells were washed 3 times with washing buffer and captured FXIIIa was quantified subsequent to addition of 100 µL of 25 µM FXIIIa-specific fluorogenic substrate at λ_ex_ 310 nm and λ_em_ 420 nm using the Synergy 2 microplate reader.

### 2.8. Interference of Aptamers on Fibrinogen Binding of FXIIIa and Alpha2-Antiplasmin (α2AP) Incorporation

Initially, white maxisorb microtiter plates were coated overnight at 4 °C with 3 µM fibrinogen (Hemocomplettan^®^P, 100 µL/well) in coating buffer followed by blocking the remaining binding sites using 200 µL of blocking buffer for each well. FXIIIa (25 nM) was incubated for 30 min at room temperature (RT) with increasing concentrations of 3′-biotinylated aptamers (1–316 nM) in binding buffer. Then, 100 µL of the mixture was transferred to the fibrinogen-coated wells. After 30 min of incubation, the wells were washed three times using washing buffer and the fibrinogen bound FXIIIa was quantified using the fluorogenic substrate (25 µM in substrate buffer, 100 µL/well, λ_ex_ 310 nm and λ_em_ 420 nm). 

The same experiment has been performed with minor changes to confirm α2AP incorporation to fibrin monomers coated to the wells. Briefly, 25 µL of FXIIIa (48 nM) was mixed with 25 µL of 400 nM of each aptamer in reaction tube. After 30 min of incubation at RT, the reaction mixture was transferred to the fibrinogen-coated plates followed by addition of 25 µL of 2.8 nM thrombin and 25 µL of 570 nM α2-AP. After another 30 min of incubation, the wells were washed three times using automated plate washer and 100 µL of peroxidase conjugated goat anti-human α2AP antibody (diluted 1:2000 in assay buffer) was added to each well. The incorporation of α2AP was quantified after washing and addition of 100 μL/well BM chemiluminescence substrate and luminescence measurement using the Synergy 2 microplate reader.

### 2.9. Rotational Thromboelastometry Analysis (ROTEM) 

Rotational thromboelastometry was performed on ROTEM^®^ delta system (TEM Innovations GmbH; Munich, Germany) using citrated pool plasma (CPP; in house preparation) or citrated whole blood. In brief, the extrinsic pathway of coagulation was activated by the addition of a calcium chloride solution (0.2 M) containing 6.25% (v/v) tissue factor (Innovin^®^). In order to assess the impact of aptamers on the kinetics of clot formation, CPP or whole blood was spiked with aptamers or controls to reach final concentrations of 2 µM. Clot formation was monitored in terms of ROTEM key parameters such as clotting time (CT), clot formation time (CFT), maximum clot firmness (MCF), and maximum clot elasticity (MCE). All tests were performed in triplicate. Tridegin and a non-binding negative control oligonucleotide were used as positive and negative controls, respectively. 

To evaluate the impact of aptamers on clot lysis, CPP was spiked first with tissue-type plasminogen activator (tPA, 125 ng/mL final concentration) and then with aptamers or controls to reach the final concentration of 2 µM. The coagulation was activated by the addition of a calcium chloride solution (0.2 M) containing 1% (*v/v*) tissue factor. Clot formation was monitored for 60 min to obtain the fibrinolysis-dependent key parameters such as fibrinolysis index after 30 min (LI30) and lysis onset time (LOT). All tests were performed in triplicate. Tridegin and a non-binding negative control oligonucleotide were used as positive and negative controls, respectively.

### 2.10. Molecular Modeling of FXIIIa-Aptamer Binding

In order to structurally explore the putative binding modes of the aptamers to the FXIIIa, a modeling/docking study was performed. All described aptamers were structurally modeled using a commonly used RNA-DNA adaptive modeling strategy [29]. Briefly, secondary structures from identified ssDNA-sequences were built and equivalent 3D ssRNA models were constructed. The equivalent ssRNA sequence and its secondary structure predicted from the Mfold server were submitted to the 3dRNA v2.0 structure modeling server under default conditions [30,31]. The highest scoring ssRNA models for each aptamer were then transformed into ssDNA 3D structures by importing the structures into YASARA [32]. In YASARA, hydrogen atoms were added and the H5 atom of each Uracil residue was replaced with a methyl group and the ribose sugar backbone was replaced with deoxyribose. The charges were assigned based on the AUTOSMILES function embedded in YASARA. The PDB co-ordinates of these aptamer ssDNA structural models were used to dock onto the crystal structure co-ordinates of FXIIIa on the docking server Hdock (http://hdock.phys.hust.edu.cn/, accessed on 9 February 2021) [33]. The structure of FXIIIa that was used as the receptor in the docking simulations was obtained in a pre-equilibrated state from the MD trajectory of the crystal structure of FXIIIa (PDB: 4KTY) reported in an earlier study [34,35]. The highest scoring docking poses for each aptamer was critically analyzed with respect to previously suggested fibrinogen (mainly alpha chain) and α2AP binding site residues as well as catalytically important active site residues [36]. Electrostatic surface potential of the FXIIIa was calculated and graphically depicted using the Adaptive Poisson-Boltzmann Solver integrated within YASARA.

### 2.11. Statistical Analysis

IC_50_ values were calculated by a log (inhibitor) vs. response-variable slope (four parameters) curve fit. The clot formation parameters in plasma spiked with aptamers were compared with controls using one-way ANOVA with Tukey post-test. All calculations were done by using GraphPad Prism version 8.3.0 for windows.

## 3. Results

### 3.1. Aptamer Selection and Characterisation

Using CE-SELEX and NGS, six aptamers against FXIIIa were identified and named FA1, FA2, FA3, FA6, FA8 and FA12. The aptamers showed high binding affinities to FXIIIa in the nanomolar range (K_D_ values range from 50 nM to 94 nM) and only low affinities to FXIII or FXIII-A were observed (Table 1). Multiple sequence alignment showed no consensus motif among the different aptamer sequences.

### 3.2. Impact of Aptamer Binding on Isopeptidase and Transglutaminase Activity of FXIIIa

**Figure 1:**
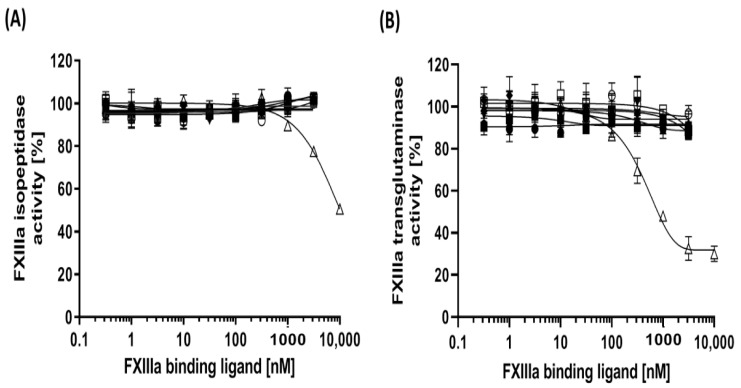
Effect of the FXIIIa-binding aptamers on FXIIIa catalytic activity. (**A**) Isopeptidase acitivity. Hydrolysis rates of the peptide substrate Abz-NE(CAD-DNP)EQVSPLTLLK-OH was measured at λ_ex_ 310 nm and λ_em_ 420 nm after incubation of FXIIIa (12.8 nM) with increasing concentrations of aptamers or controls. (**B**) Transglutaminase activity. FXIIIa (4 nM) was incubated with increasing concentrations of aptamers or controls and reacted with a synthetic peptide substrate to finally measure the reduction of absorbance at 340 nm according to released ammonia and decreased NADH. Data are shown as means of triplicates ±SD. ● FA1, ■ FA2, ▲ FA3, ▼ FA6, ♦ FA8, ○ FA12, □ negative control sequence, ∆ Tridegin.

The isopeptidase activity of FXIIIa was measured using a fluorogenic peptide substrate, Abz-NE(CAD-DNP)EQVSPLTLLK-OH. FXIIIa-dependent hydrolysis of an isoamide bond linking a glutamine residue to a synthetic fluorescent quencher unmasks the fluorescence of the fluorophore linked to the synthetic glutamine-containing peptide, which was detected spectrofluorometrically using excitation (λ_ex_) and emission (λ_em_) wavelengths of 310 nm and 420 nm, respectively [37]. The isopeptidase activity of FXIIIa remained unaffected by increasing concentrations of the aptamers (Figure 1A, Table 2). Furthermore, to evaluate the modulation of transglutaminase activity of FXIIIa subsequent to aptamer binding, FXIIIa was incubated with the same aptamer concentrations used in isopeptidase activity test and the mixtures were subjected to corresponding activity measurement proportional to ammonia release during a transglutaminase reaction and quantification of ammonia-dependent oxidation of NADH [37]. A slight reduction in transglutaminase activity of FXIIIa after incubation with high concentrations of FXIIIa-specific aptamers comparable to the negative control was observed, whereas the most noticable reduction was observed as 12.5% to 12.9% after incubation of FA6 and FA2 with FXIIIa, respectively (Figure 1B, Table 2). In both test systems, tridegin was used as positive control. The isopeptidase activity of FXIIIa was only partially inhibited by 50% even at high concentrations of tridegin (12 µM). The same concentration range of tridegin inhibited the transglutaminase activity of FXIIIa more efficiently and the IC_50_ value was calculated as 1.68 µM (95% confidence interval [CI]: 1.36 µM to 2.1 µM).

### 3.3. Determination of Capturing Capacity of Aptamers

**Figure 2:**
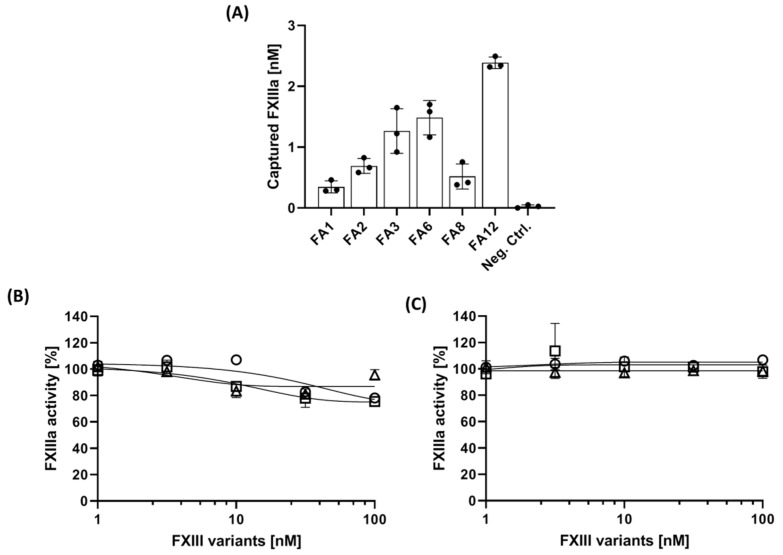
Determination of the capacity of aptamers in FXIIIa capturing. (**A**) FXIIIa (30 nM) was incubated in microtiter plates primed with each aptamer. Captured FXIIIa was quantified using the FXIIIa-specific fluorogenic peptide substrate. Subsequently, FXIIIa-capturing by the FA6 ap-tamer (**B**) and the FA12 aptamer (**C**) in the presence of increasing concentrations of FXIII, FXIII-A, and FXIII-B was determined. All results are shown as means with standard deviations of tripli-cates. ○ FXIII, □ FXIII-A, ∆ FXIII-B.

The capability of immobilized aptamers to capture FXIIIa from purified system was tested using microtiter plates primed with 3′-biotinylated aptamers. All aptamers were able to capture FXIIIa albeit on different levels. According to Figure 2A, the immobilized FA12 aptamer captured 10% (3 nM) of FXIIIa from starting solution containing 30 nM FXIIIa after 30 min of incubation. This value reduced to 5% (1.3 to 1.5 nM) using FA3 and FA6 and to 1.6% (less than 0.5 nM) when using FA1, FA2, and FA8 as capturing ligands. Performing the assay in wells primed with the non-specific oligonucleotide sequence excluded unspecific binding of FXIIIa. The two most efficient aptamers, FA6 and FA12 were subjected to the next experiment to evaluate their capturing ability in the presence of other FXIII subunits. As could be expected according to determined binding affinities (cf. Table 1), the results shown in Figure 2B,C demonstrated an only limited impact of the presence of FXIII, FXIII-A or FXIII-B on FXIIIa-binding to the aptamers. The overall results suggested FA12 as the most efficient capturing ligand for FXIIIa.

### 3.4. The Impact of Aptamer on Fibrinogen-Binding of FXIIIa

**Figure 3:**
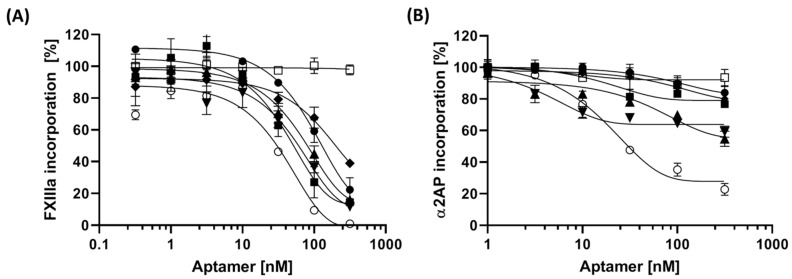
Aptamer binding reduced incorporation of FXIIIa and α2-antiplasmin (α2AP) to fibrinogen. Retention of (**A**) FXIIIa or (**B**) α2AP in fibrinogen coated microtiter plates in the presence of increasing concentrations of aptamers was detected using fluorogenic peptide substrate or a polyclonal antibody against α2AP, respectively. All results are shown as means with standard deviations of triplicates. ● FA1, ■ FA2, ▲ FA3, ▼ FA6, ♦ FA8, ○ FA12, □ negative control sequence.

Binding of FXIIIa to fibrinogen-primed microtiter plates was quantified in the presence of increasing concentrations of aptamers. The data showed that all aptamers could interfere with binding of FXIIIa to fibrinogen albeit at different levels (Figure 3A). The aptamer concentrations which could lower fibrinogen-binding of FXIIIa by 50% (IC_50_) ranged from 38 nM to 136 nM. Among all aptamers, with IC_50_-values of ~40 nM, FA2 and FA12 inhibited FXIIIa binding most efficiently (Table 2). 

It is well established that in the presence of FXIIIa, α2AP becomes ligated to the distal α chains of fibrin or fibrinogen [38]; therefore any interference of FXIIIa incorporation in fibrinogen would affect the fibrinogen-α2AP incorporation as well. To detect that, different concentrations of aptamers were incubated with FXIIIa and α2AP in fibrinogen-coated plates and the amount of immobilized α2AP was detected using a peroxidase conjugated anti-human α2AP antibody. Among all aptamers, FA6 showed the lowest IC_50_ of 4.3 nM but the maximum achievable reduction in α2AP incorporation using FA6 was only ~40%. By using FA12, although at a higher IC_50_–value of 17 nM, the total reduction in α2AP incorporation was found to be ~80% which, again, demonstrated the highest overall efficiency of FA12. (Figure 3B, Table 2).

### 3.5. Influence of Aptamers on Clot Formation as Assessed by ROTEM-Analysis

To evaluate the impact of the aptamers (2 µM final concentration) on clot formation and firmness in plasma, thromboelastometry was used. Among all aptamers, FA2 and FA12 showed a reduction in maximum clot firmness (MCF) and reduced amplitudes after 10 min (A10) and 20 min (A20) when compared to the plasma sample spiked with the negative control sequence (Table 3 and Supplementary Materials Figure S1). The maximum clot elasticity (MCE), which is a function of MCF, was found to be reduced from 25 in plasma samples spiked with the negative control to 14 and 19 in plasma samples spiked with FA2 and FA12, respectively. Furthermore, spiking the plasma samples with each FXIII-binding aptamer prolonged the clot formation time (CFT) to more than 2700 s (45 min) which was not seen in plasma spiked with negative control sequence. The maximum amplitude after 10 min and 20 min (A10 and A20) were reduced significantly by using FA2 (*p* < 0.0001), FA8 (*p* < 0.05), and FA12 (*p* < 0.001 for A10 and *p* < 0.01 for A20). Interestingly, addition of tridegin to the plasma sample (2 µM final concentration) could reduce A10 and A20 amplitudes significantly (in comparison to non-spiked pool plasma), but was unable to affect the maximum clot firmness and elasticity. When using citrated whole blood, however, the detectable impact of aptamers on clot formation as well as clot firmness was found to be strongly reduced. Nevertheless, spiking of whole blood with FA12 significantly influenced MCE-, A10-, A20-, and MCF-values. The same effects were found for aptamers FA3 and FA8 albeit at lower level (Supplementary Materials, Figure S2 and Supplementary Materials, Table S1). In order to monitor the impact of aptamers on fibrinolysis and the corresponding key parameters of finrionlysis such as lysis onset time (LOT), citrated pool plasma was spiked in advance with tPA. Data showed that FA2 is able to reduce LOT. However, as lysis started after 12 to 19 min, A20 which might be considered as a function of lysis and lysis index after 30 min (LI30) were affected negatively by using FA2 (Supplementary Materials, Figure S3 and Supplementary Materials, Table S2). The minor changes in A20-, LI30-, and LOT-values which were observed after addition of other aptamers to the pool plasma were not statistically significant.

### 3.6. Aptamer Binding Poses on FXIIIa and Implications for Substrate Access

**Figure 4:**
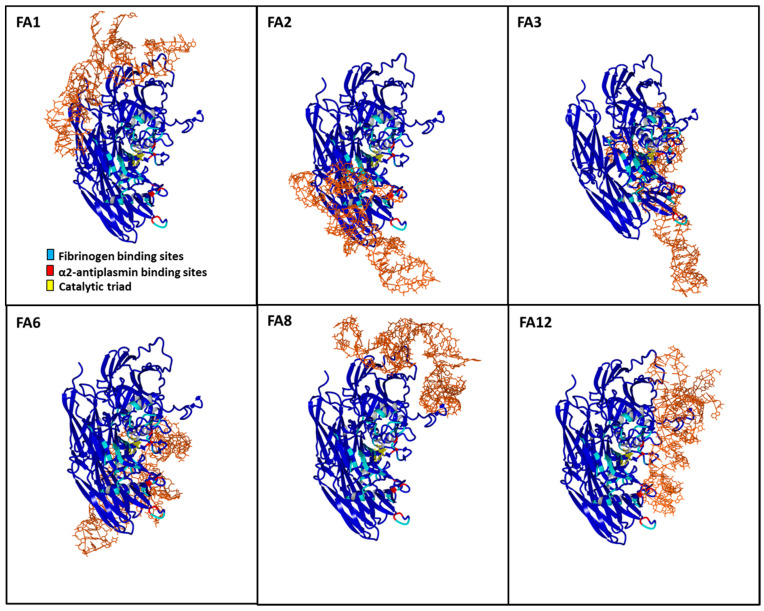
Aptamer docking on FXIIIa. The highest scoring docking pose for each of the six aptamers on FXIIIa is illustrated. The aptamer models are colored brown and represented in stick format while the FXIIIa backbone is represented in blue colored ribbon format. The fibrinogen and α2AP binding sites on FXIIIa, based on a previous docking study have been colored cyan and red, respectively [36]. The catalytic triad residues on FXIIIa are colored yellow.

The electrostatic surface of FXIIIa can be split into major surfaces (Supplementary Materials Figure S4). The surface bearing the catalytic site residue primarily bears a negative charge while the surface behind this region carries a positive charge. In a majority of docking poses for aptamers FA1, FA3, FA6, and FA8, the DNA appears to be anchored by a major positive electrostatic patch behind the cluster of catalytic residues and oriented themselves along the different domains of FXIIIa (Figure 4). In the case of aptamers, FA2 and FA12, however, the docking poses did not anchor around the major positive electrostatic patch and also showed an overlap of the predicted ssDNA-structure with the fibrinogen and/or the α2AP binding sites on FXIIIa. The binding sites of FA2 and FA12 were also found to be in close proximity to the catalytic residues of FXIIIa. However, only FA12 showed binding sites that overlap with both, the fibrinogen as well as the α2AP binding site on FXIIIa. In contrast to FA2 and FA12, none of the aptamers FA1, FA3, FA6, and FA8 were observed to be either overlapping or significantly binding close to the fibrinogen- or α2AP-binding site or the catalytic residues of FXIIIa. Although these aptamers are not expected to physically interfere with the fibrinogen or α2AP binding sites, an allosteric influence on substrate binding cannot be excluded.

## 4. Discussion

Aptamers can be selected from randomized oligonucleotide libraries to bind virtually to any protein of interest. Here, we describe for the first time the selection and characterization of a group of FXIIIa-binding DNA aptamers. Employing capillary electrophoresis for efficient exclusion of non-binding ssDNA-molecules favored the selection of aptamers binding to FXIIIa with high affinity and specificity [26,27]. Functional characterization of the identified aptamers demonstrated that these molecules do not interfere with the isopeptidase or transglutaminase activity of FXIIIa as measured using small peptide substrates, which means that none of these aptamers directly blocks the active site of the enzyme. In addition, aptamer binding does not induce conformational changes leading to altered accessibility of the active site. One of these aptamers, named FA12, was able to capture efficiently FXIIIa even in the presence of other FXIII subunits at physiological concentrations. Indeed, FXIIIa captured by immobilized FA12 could be easily detected and quantified by hydrolysis rates of a fluorogenic isopeptidase substrate. These features render the FA12 aptamer as a diagnostic tool in situations where the capturing and detection of the active enzyme may be of interest. 

Selected aptamers showed at least 5- to 10-fold reduced affinity for zymogenic FXIII as well as FXIII-A. These characteristics suggest that the aptamer binding sites on FXIIIa are located on (neo)epitopes that only exposed upon FXIII-activation and/or FXIII-B subunits dissociation. The surface electrostatic distribution on the zymogenic FXIII-A dimeric structure is far more heterogeneous than that of FXIIIa (Supplementary Materials, Figure S5). FXIIIa shows specific negative or positive electrostatic patches on its surface, indicative of the formation of substrate binding sites. The lack of such major electrostatic patches on zymogenic FXIII-A may be the reason for the low affinity of the aptamers to the same.

It is well established that in the presence of thrombin-activated FXIIIa, α2AP becomes ligated to the distal Aα chains of fibrin(ogen) at lysine 303 (two potential sites per molecule) and only after it has been incorporated into fibrin(ogen) via FXIIIa, is α2AP an effective inhibitor of fibrinolysis. Therefore, FXIIIa not only crosslinks fibrin chains for clot stabilization, but also contributes to clot stiffness by crosslinking of other plasma proteins involved in clot formation and fibrinolysis to fibrin(ogen) [38,39]. Hence, the impact of an inhibitor ligand might be applied directly through the modulation of the enzyme active site or through the modification of enzyme-binding and/or incorporation of the natural enzyme substrates. Our data show that all aptamers inhibit FXIIIa-fibrin(ogen) interaction while surprisingly, only FA12 caused a significant dose-dependent decrease in the incorporation of α2AP to fibrinogen. This finding correlates with the study of Smith et al., where inhibition of the FXIIIa-fibrin αC interaction using a synthetic peptide caused a dose-dependent reduction in the incorporation of α2AP to fibrin [40]. Published literature on FXIII-fibrinogen or FXIII-α2AP interaction is available in abundant amount but not in structural atomistic detail. Residue-wise interaction details are better characterized for the fibrinogen and α2AP interaction interfaces than for FXIII. Key residues and regions on the αC region and the γ chain of fibrin(ogen) involved in the FXIII-fibrinogen interaction are well known [41]. Specific details of FXIII interfaces are limited to only few handful studies and these studies are also limited in their exactness [36,40]. Most of these studies present only one dimensional aspect of these interactions, primarily because of the presence of multiple reactive glutamine donor residues on large substrates like fibrinogen [42]. Furthermore, these studies are heavily reliant on experimentally driven modeling approaches that leave room for other possibilities. The structure–function correlations determined in our study by combining modeling/docking strategies with quantitative in vitro assays result is in line with most of the structural detail currently available regarding FXIII-fibrinogen or FXIII-α2AP interactions. High scoring docking poses of only selected aptamer models on the FXIIIa structure, namely FA2 and FA12, were observed to have spatial overlap with the binding sites of the FXIII substrates, fibrinogen and α2AP. More specifically, it is only FA12 that has predicted binding sites overlapping with both, fibrinogen and α2AP, while predicted FA2 binding sites only overlap with those of fibrinogen. As mentioned earlier, FA12 is the only aptamer that shows a relevant dose-dependent reduction in the incorporation of α2AP to fibrin(ogen). One characteristic of the FA2 and FA12 docking poses is that although they do occupy specific substrate binding sites, the docking orientation leaves room for smaller substrates to access the catalytic site. Transition state intermediate models of FXIII activation have shown that a hydrophobic tunnel formed by the Trp279 and Trp379 residues and an oxyanion hole at the bottom of the catalytic pocket are key to the stabilization of substrate-enzyme intermediates [34,43]. Therefore, small substrates like peptides which can be stabilized within this hydrophobic tunnel without the aid of neighboring binding sites can successfully circumvent the competitive inhibitory effect of FA2 and FA12 aptamers. Since the isopeptidase or transglutaminase assays employ small peptide substrates, none of our aptamers, including FA2 and FA12, show any inhibitory effect in these assays. The remaining aptamers show no direct specificity for fibrinogen and α2AP binding sites but as our functional data suggests, they might still maintain allosteric control over fibrinogen binding and subsequent cross-linking. These aptamers are observed to bind to counter-surfaces around regions which have been demonstrated to affect substrate binding for e.g., calcium binding sites (Supplementary Materials, Figure S6) [44].

In addition, thromboelastometry results obtained from plasma samples spiked with aptamers showed reduction in both amplitudes of clot formation and clot firmness in the presence of aptamers FA2 and FA12. Furthermore, FA2 showed promising effect on reduction of lysis onset time, although these effects were found to be less pronounced when using whole blood, these results indicate that, indeed, only direct interaction with the fibrinogen and/or a2AP binding sites of FXIIIa leads to efficient reduction of clot formation.

Taken together, the presented data demonstrate that inhibition of binding of FXIIIa to its natural substrates, fibrinogen and α2AP by DNA-aptamers can be used to reduce clot firmness and to increase susceptibility of the fibrin clot to fibrinolysis. These features especially render the aptamer FA12 as a potential anticoagulant drug candidate molecule. Furthermore, it could be shown that FA12 also represents a candidate ligand for the development of a FXIIIa (enzyme) capture assay.

## Figures and Tables

**Table 1:** Oligonucleotide sequence and binding affinities of aptamers to different subunits of FXIII.

Aptamer	Oligonucleotide Sequence (5′–3′)	Binding Affinity (K_D_, nM)		
		FXIIIa	FXIII-A	FXIII
FA1	**AAGCAGTGGTAAGTAGGTTGA**CCATCCGCCGATTAGGCAATCACGTGGCTTAGGCACCGTA**TCTCTTCGAGCAATCCACAC** *	50.4	>1000	>500
FA2	**AAGCAGTGGTAAGTAGGTTGA**GCTTTCATCACCCGTCGGTTAGACTAAGTCTCCAGCATCC**TCTCTTCGAGCAATCCACAC**	79.1	>1000	>1000
FA3	**AAGCAGTGGTAAGTAGGTTGA**CCATCGTAGTTCGTACACCAGTCCATTCTAGGACTCACAA**TCTCTTCGAGCAATCCACAC**	92.9	>1000	>1000
FA6	**AAGCAGTGGTAAGTAGGTTGA**CGCTGCGGGCATCACGTAAGTTAATATATCACTCGGCTAT**TCTCTTCGAGCAATCCACAC**	93.5	>1000	>1000
FA8	**AAGCAGTGGTAAGTAGGTTGA**TCATCATGTTATTTTTGTTCCATCACGCCAGTTCTGGTTC**TCTCTTCGAGCAATCCACAC**	79.1	>1000	>1000
FA12	**AAGCAGTGGTAAGTAGGTTGA**TCATCCTCTTTAAGTCATCACTTTAGTTTCTCCATCTACA**TCTCTTCGAGCAATCCACAC**	51.8	>1000	>500

* The fixed primer binding sites are indicated in bold font.

**Table 2:** Key characteristics of FXIIIa aptamers.

Aptamer	FXIIIa Isopeptidase Activity (Max. Inhibition) ^1^	FXIIIa Transglutaminase Activity (Max. Inhibition) ^1^	IC_50_ (nM) of Inhibition of FXIIIa Incorporation to Fibrinogen	IC_50_ (nM) of Inhibition of α2AP Incorporation to Fibrinogen
FA1	<1%	9.34%	89.51 (53.3–194) ^2^	86.39 (35–266)
FA2	<1%	12.87%	39.27 (24.65–63.1)	17.52 (9–42.7)
FA3	<1%	11.1%	66.15 (43.05–109.5)	60.32 (24.3–164)
FA6	<1%	12.47%	57 (34.6–98.6)	4.28 (2.9–6.5)
FA8	<1%	8.3%	136.3 (60.6–n.d.)	88.9 (36–159)
FA12	<1%	3.38%	37.95 (22.6–64.6)	17.4 (13.2–23.3)
Neg. Ctrl.	<1%	11.35%	Not affected	Not affected

^1^ The maximum inhibition was shown in the presence of highest concentration of aptamer (3.16 µM); ^2^ Data showed in parenthesis represents 95% confidence interval.

**Table 3:** Results of rotational thromboelastometry (ROTEM) analysis of plasma samples spiked with 2 µM of different aptamers or tridegin.

	CT (s)	CFT (s)	MCF (mm)	A10 (mm)	A20 (mm)	MCE
Pool plasma	58.33 ± 6.03	716.33 ± 184	22 ± 1	19.67 ± 0.58	21.67 ± 0.58	28.22 ± 1.64
FA1	48 ± 4.58	>2700	17.67 ± 2.08	16.33 ± 1.53	17.33 ± 1.53	21.51 ± 3.11
FA2	55.67 ± 5.51	>2700	12 ± 1 ****	11.33 ± 0.58 ****	12 ± 1 ****	13.65 ± 1.29 ****
FA3	53.67 ± 1.15	>2700	19.33 ± 0.58	17 ± 1	19 ± 1	23.97 ± 0.89
FA6	55.50 ± 2.12	>2700	19.5 ± 0.51	17.5 ± 0.71	19.5 ± 0.71	24.23 ± 1.09
FA8	60.33 ± 5.03	>2700	17.33 ± 0.58	15.67 ± 0.58 *	17 ± 0 *	20.97 ± 0.85
FA12	62.33 ± 4.51	>2700	16 ± 1 **	14.33 ± 0.58 ***	15.67 ± 1.15 **	19.06 ± 1.42 **
Tridegin	60.67 ± 2.1	2396 ± 74	19.67 ± 1.5	15.67 ± 0.58 ***	17.67 ± 0.58 ***	24.5 ± 2.35
Neg. Ctrl.	54.3 ± 4.04	918.5 ± 105	20 ± 1.73	18.3 ± 1.15	19.66 ± 1.53	25.03 ± 2.67

CT, clotting time; CFT, clot formation time; MCF, maximum clot firmness; A10, amplitude after 10 min; A20, amplitude after 20 min; MCE, maximum clot elasticity. Data are shown as mean ± SD of three independent measurements. All aptamers were compared to the negative control sequence whereat tridegin was compared to non-spiked pool plasma using one-way ANOVA test. * *p* < 0.05, ** *p* < 0.01, *** *p* < 0.001, **** *p* < 0.0001.

## Data Availability

The data presented in this study are available on request from the corresponding author.

## Supplementary Materials

The following are available online at https://www.mdpi.com/2077-0383/10/4/677/s1, Figure S1: The thromboelastogram resulted from citrated pool plasma spiked with (A) FA1, (B) FA2, (C) FA3, (D) FA6, (E) FA8, (F) FA12, (G) negative control sequence, (H) no aptamer and (I) tridegin to reach the final concentration of 2 µM for aptamers or tridegin. Plasma samples were spiked with aptamers, negative control sequence or tridegin and throboelastography was started by addition of 0.2 M CaCl2 solution containing 6.25% recombinant tissue factor; Figure S2: The thromboelastogram resulted from citrated blood spiked with (A) FA1, (B) FA2, (C) FA3, (D) FA6, (E) FA8, (F) FA12, (G) negative control sequence, (H) no aptamer and (I) tridegin to reach the final concentration of 2 µM for aptamers or tridegin. Throboelastography was started by addition of 0.2 M CaCl_2_ solution containing 6.25% recombinant tissue factor; Figure S3: The thromboelastogram resulted from citrated pool plasma spiked first with 125 ng/mL tissue-type plasminogen activator and then with (A) FA1, (B) FA2, (C) FA3, (D) FA6, (E) FA8, (F) FA12, (G) negative control sequence, (H) no aptamer and (I) tridegin to reach the final concentration of 2 µM for aptamers or tridegin. Throboelastography was started by addition of 0.2 M CaCl_2_ solution containing 6.25% recombinant tissue factor; Figure S4: The image shows a PBS-based electrostatic surface representation of the FXIIIa structure from structurally reverse orientations. Red color indicates negative surface electrostatic potential, whereas blue represents positive potential; Figure S5: Zymogenic FXIIIA_2_ vs. activated FXIII electrostatic surface. The zymogenic FXIIIA_2_ (PDB ID: 1f13) and activated FXIIIa (PDB ID: 4kty) crystal structures are depicted by their PBS based electrostatic molecular surface representation. Red color indicates negative surface electrostatic potential, whereas blue color represents positive potential; Figure S6: A close-up view of the FA6 aptamer docking pose on FXIIIa. The aptamer is depicted in black stick format while on a PBS based molecular surface representation of FXIIIa is shown. Red color indicates negative surface electrostatic potential, whereas blue represents positive potential. The charged residues of calcium binding site 1 of FXIIIa are depicted in blue colored stick format [43]; Table S1: Results of ROTEM analysis of citrated whole blood spiked with 2 µM of different aptamers or controls; Table S2: Results of ROTEM analysis of citrated pool plasma spiked with 125 ng/mL tissue-type plasminogen activator and 2 µM of different aptamers or controls.
