# Supplementary material for: Functional and Structural Characterization of Nucleic Acid Ligands That Bind to Activated Coagulation Factor XIII
Source: J Clin Med. 2021 Feb 10;10(4):677. doi: 10.3390/jcm10040677 (PMC7916480; doi:10.3390/jcm10040677)
Supplement: Supplementary file 1 [file jcm-10-00677-s001.pdf]

Journal of
Clinical Medicine

MDPI

Supplementary Materials:

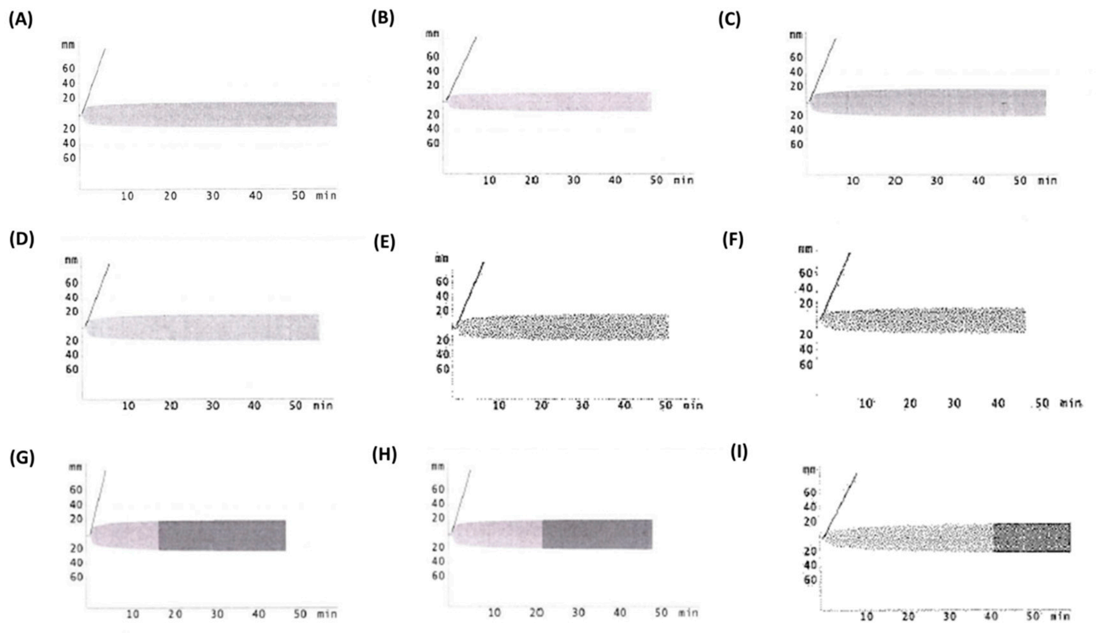

Supplemntary Figure 1. The thromboelastogram resulted from citrated pool plasma spiked with (**A**) FA1, (**B**) FA2, (**C**) FA3, (**D**) FA6, (**E**) FA8, (**F**) FA12, (**G**) negative control sequence, (**H**) no aptamer and (**I**) tridegin to reach the final concentration of 2 μM for aptamers or tridegin. Plasma samples were spiked with aptamers, negative control sequence or tridegin and throboelastography was started by addition of 0.2 M $CaCl_2$ solution containing 6.25% recombinant tissue factor.

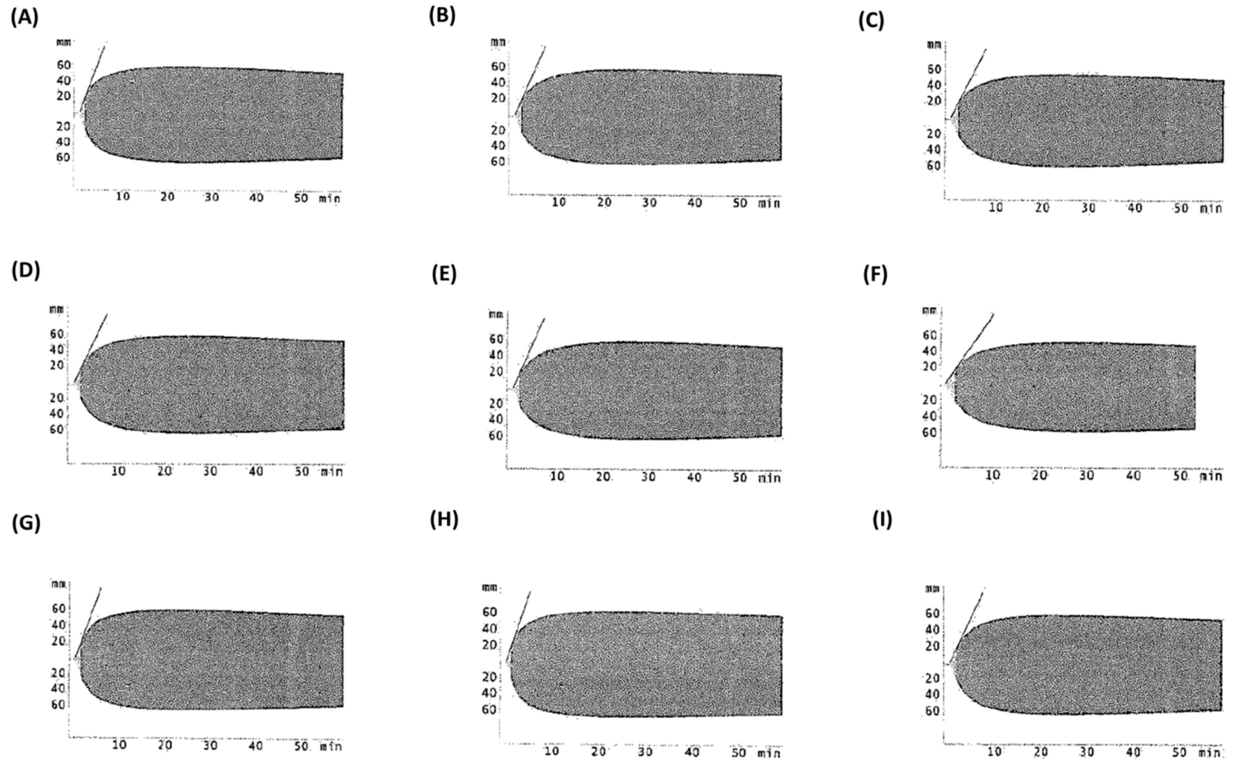

Supplemntary Figure 2. The thromboelastogram resulted from citrated blood spiked with (**A**) FA1, (**B**) FA2, (**C**) FA3, (**D**) FA6, (**E**) FA8, (**F**) FA12, (**G**) negative control sequence, (**H**) no aptamer and (**I**) tridegin to reach the final concentration of 2 μM for aptamers or tridegin. Throboelastography was started by addition of 0.2 M $CaCl_2$ solution containing 6.25% recombinant tissue factor.

Supplementary Table 1. Results of ROTEM analysis of citrated whole blood spiked with 2 μM of different aptamers or controls.

	CT [s]	CFT [s]	MCF [mm]	A10 [mm]	A20 [mm]	MCE
**Whole blood**	80.33 ± 6.67	60.67 ± 6.03	66.33 ± 1.15	61 ± 1	65.67 ± 0.58	197.26 ± 9.99
**FA1**	80.67 ± 0.58	66.67 ± 6.03	63.33 ± 0.58	57.67 ± 0.58	63.33 ± 0.58	172.77 ± 4.33
**FA2**	87.33 ± 1.53	84 ± 2.65	60 ± 1	53.67 ± 0.58	59.67 ± 0.58	150.1 ± 6.25
**FA3**	77.33 ± 2.52	91.33 ± 3.79	58.67 ± 1.15**	52.33 ± 1.53*	58.33 ± 0.58*	142.06 ± 6.88**
**FA6**	85.33 ± 8.5	79.33 ± 5.86	62.67 ± 0.58	56 ± 1	62.33 ± 0.58	167.9 ± 4.1
**FA8**	79.67 ± 7.5	108.33 ± 31.78*	60 ± 2.65	51 ± 4.36**	58.67 ± 3.21*	150.71 ± 16.08
**FA12**	68.33 ± 3.85*	109.66 ± 14.36*	56.67 ± 0.58***	49.67 ± 1.53**	56.33 ± 1.15***	130.8 ±3.05***
**Tridegin**	89.67 ± 3.75	88 ± 16.52	61.67 ± 1.53**	55.67 ± 2.52	61.67 ± 1.53*	161.14 ± 10.28**
**Neg. Ctrl.**	87.33 ± 8.62	69.33 ± 3.79	63.33 ± 0.57	58 ± 1	63 ± 1	172.77 ± 4.33

CT, clotting time; CFT, clot formation time; MCF, Maximum clot firmness; A10, amplitude after 10 min; A20, amplitude after 20 min; MCE, maximum clot elasticity. Data are shown as mean ± SD of three independent measurements. All aptamers were compared to the negative control sequence whereat tridegin was compared to non-spiked whole blood using one-way ANOVA test. * $p < 0.05$, ** $p < 0.01$, *** $p < 0.001$.

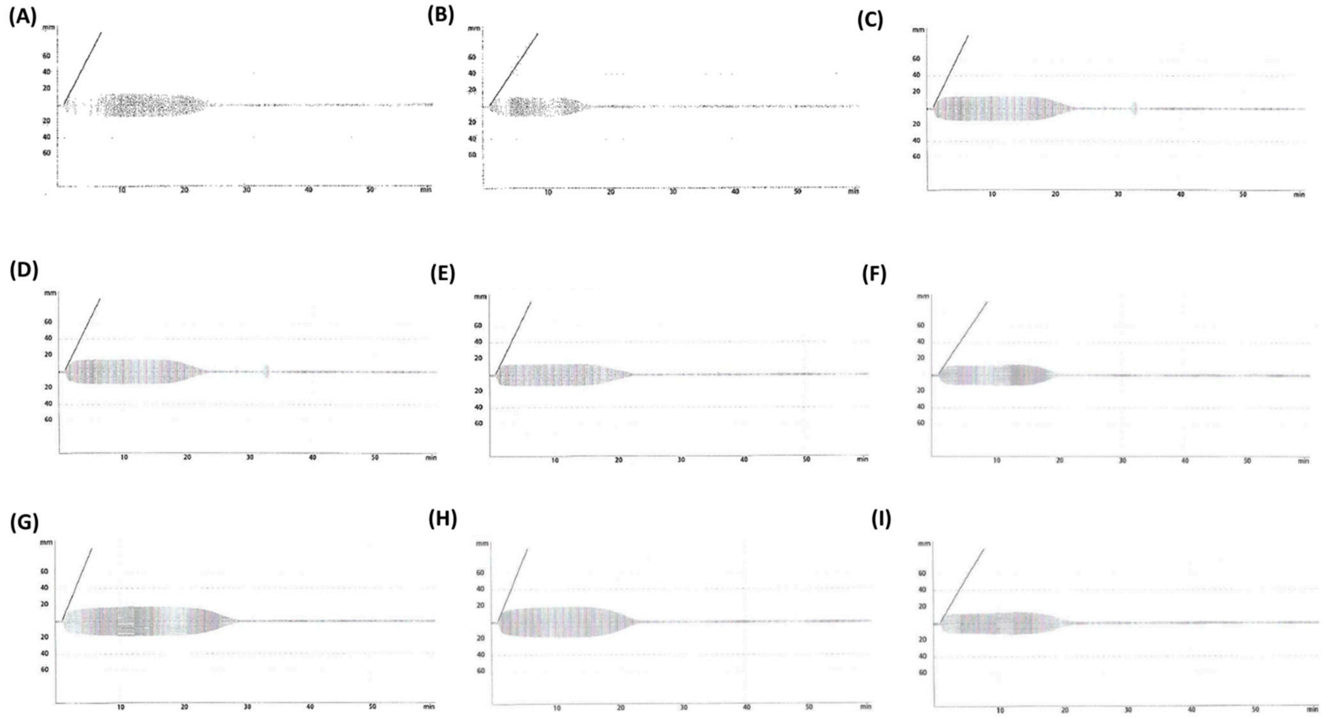

Supplemntary Figure 3. The thromboelastogram resulted from citrated pool plasma spiked first with 125 ng/mL tissue-type plasminogen activator and then with (**A**) FA1, (**B**) FA2, (**C**) FA3, (**D**) FA6, (**E**) FA8, (**F**) FA12, (**G**) negative control sequence, (**H**) no aptamer and (**I**) tridegin to reach the final concentration of 2 µM for aptamers or tridegin. Throboelastography was started by addition of 0.2 M $CaCl_2$ solution containing 6.25% recombinant tissue factor.

Supplementary Table 2. Results of ROTEM analysis of citrated pool plasma spiked with 125 ng/mL tissue-type plasminogen actiator and 2 µM of different aptamers or controls.

	A20	LI30[mm]	LOT
**Pool plasma**	2.2 ± 3.34	1.2 ± 1.79	826.2 ± 153.8
**FA1**	4.0 ± 6.1	2.33 ± 2.5	875.67 ± 248.1
**FA2**	0 ± 0*	0 ± 0*	750 ± 123.45*
**FA3**	9.5 ± 3.5	5.0 ± 4.24	1100 ± 86.27
**FA6**	1.67 ± 0.58	0.67 ± 0.58	903.33 ± 38.94
**FA8**	4.67 ± 5.0	2.33 ± 2.08	975.33 ± 162.5
**FA12**	3.0 ± 2.0	3.0 ± 4.36	943 ± 10.54
**Tridegin**	5.33 ± 2.1	1.33 ± 1.15	1004.67 ± 32.52
**Neg. Ctrl.**	12.33 ± 8.1	3.33 ± 3.06	1153 ± 179.55

MCF, Maximum clot firmness; A20, amplitude after 20 min; MCE, maximum clot elasticity; LI30, fibrinolysis index after 30 min; LOT, lysis onset time. Data are shown as mean ± SD of three independent measurements. All aptamers were compared to the negative control sequence whereat tridegin was compared to non-spiked pool plasma using one-way ANOVA test. * $p < 0.05$, ** $p < 0.01$, *** $p < 0.001$.

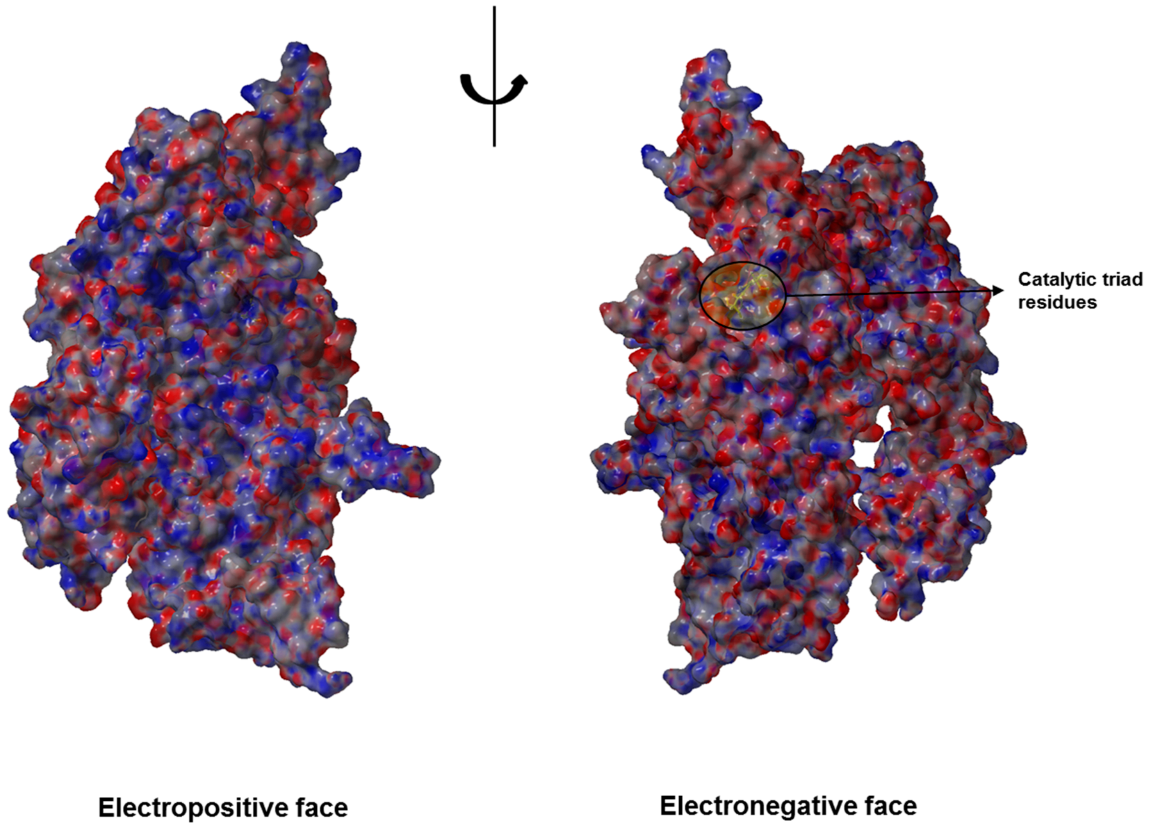

Supplementary Figure 4. The image shows a PBS-based electrostatic surface representation of the FXIIIa structure from structurally reverse orientations. Red color indicates negative surface electrostatic potential, whereas blue represents positive potential.

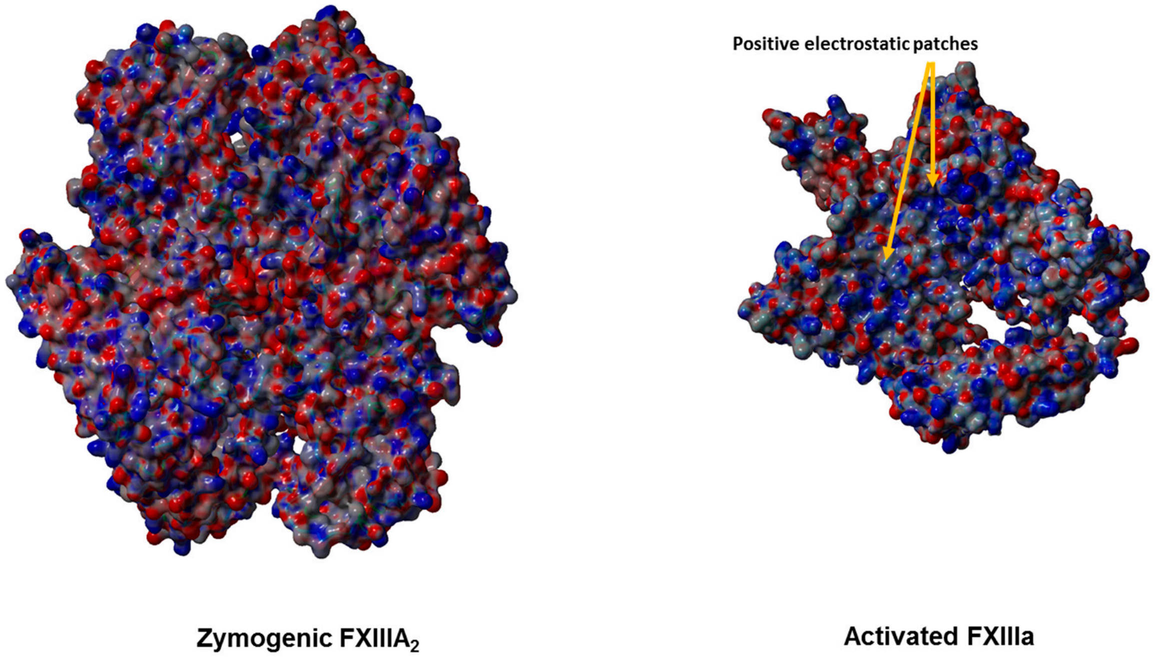

Supplementary Figure 5. Zymogenic FXIIIA2 vs. activated FXIII electrostatic surface. The zymogenic FXIIIA2 (PDB ID: 1f13) and activated FXIIIa (PDB ID: 4kty) crystal structures are depicted by their PBS based electrostatic molecular surface representation. Red color indicates negative surface electrostatic potential, whereas blue color represents positive potential.

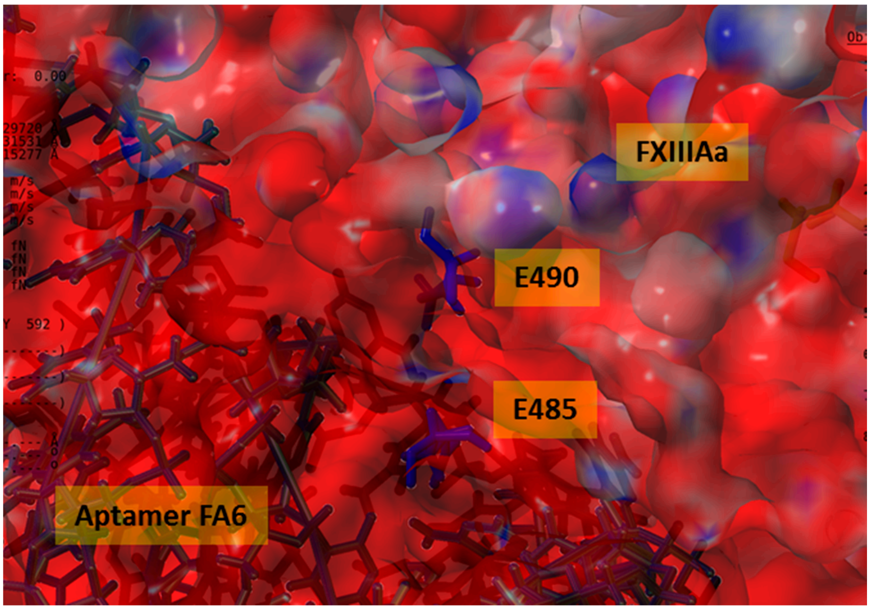

Supplementary Figure 6. A close-up view of the FA6 aptamer docking pose on FXIIIa. The aptamer is depicted in black stick format while on a PBS based molecular surface representation of FXIIIa is shown. Red color indicates negative surface electrostatic potential, whereas blue represents positive potential. The charged residues of calcium binding site 1 of FXIIIa are depicted in blue colored stick format [43].
